# Hybrid PSO-FLC for dynamic global peak extraction of the partially shaded photovoltaic system

**DOI:** 10.1371/journal.pone.0206171

**Published:** 2018-11-02

**Authors:** Hassan M. H. Farh, Ali M. Eltamaly, Mohd F. Othman

**Affiliations:** 1 Malaysia-Japan International Institute of Technology, Universiti Teknologi Malaysia, Kuala Lumpur, Malaysia; 2 Electrical Engineering Department, College of Engineering, King Saud University, Riyadh, Saudi Arabia; 3 Sustainable Energy Technologies Center, King Saud University, Riyadh, Saudi Arabia; 4 Electrical Engineering Department, Mansoura University, Mansoura, Egypt; University of Science and Technology Beijing, CHINA

## Abstract

Particle Swarm Optimization (PSO) is widely used in maximum power point tracking (MPPT) of photovoltaic (PV) energy systems. Nevertheless, this technique suffers from two main problems in the case of partial shading conditions (PSCs). The first problem is that PSO is a time invariant optimization technique that cannot follow the dynamic global peak (GP) under time variant shading patterns (SPs) and sticks to the first GP that occurs at the beginning. This problem can be solved by dispersing the PSO particles using two new techniques introduced in this paper. The two new proposed PSO re-initialization techniques are to disperse the particles upon the SP changes and the other one is upon a predefined time (PDT). The second problem is regarding the high oscillations around steady state, which can be solved by using fuzzy logic controller (FLC) to fine-tune the output power and voltage from the PV system. The new contribution of this paper is the hybrid PSO-FLC with two PSO particles dispersing techniques that is able to solve the two previous mentioned problems effectively and improve the performance of the PV system in both normal and PSCs. A detailed list of comparisons between hybrid PSO-FLC and original PSO using the two proposed methodologies are achieved. The results prove the superior performance of hybrid PSO-FLC compared to PSO in terms of efficiency, accuracy, oscillations reduction around steady state and soft tuning of the GP tracked.

## 1. Introduction

In recent years, solar energy represents one of the most important and promising renewable energy sources. It has a dramatic interest and acts as a strategic goal for many countries; due to, its clean, environmental friendly and not to mention, an inexhaustible source. In the last two decades, the leading countries in the photovoltaic (PV) area have focused on improving the PV system performance by extracting the maximum power especially under partial shading conditions (PSCs) and reducing the generated energy cost.

Under PSCs, the P-V characteristic contains multiple peaks, which are one global peak (GP) and many local peaks (LPs) for each shading pattern (SP). Most conventional maximum power point tracking (MPPT) techniques failed to track the GP. Whereas, soft computing techniques based bio-inspired such as Flower Pollination [1], Ant Bee Colony [2, 3], Firefly [4, 5], Ant Colony [6], Cuckoo Search [7], Particle Swarm Optimization (PSO) [7–13], Improved Bat [14] and S-Jaya [15] algorithms can follow the GP under the same SP. Although numerous researches [7–13] claimed that original PSO technique could track the GP generally, they neglected the fact that the SP changes from time to time; hence, the GP’s position and value also change from time to time. In addition, obvious oscillations around steady state exist with the original PSO. Therefore, it can be said that PSO can track the GP under the same SP with undesirable oscillation around steady state. In addition, PSO without certain re-initialization cannot catch the dynamic GP under time variant SPs. Instead, it sticks to the first GP caught at the beginning.

To overcome the previous mentioned drawbacks of the original PSO, Jiying *et al*. [16] proposed PSO combined with incremental conductance (IC) to reduce the steady state oscillations occurred when PSO is used alone. PSO searches for the GP area; then, IC tracks the GP under the same SP. Although, Jiying *et al*. in [16] succeeded in reducing the undesirable steady state oscillations, they considered that the SP is fixed (time invariant) and therefore, the GP is fixed too, which is impractical. Whereas, Lian *et al*. [17] proposed perturb and observe (P&O) combined with PSO (P&O-PSO) technique to track the dynamic GP under time variant SP. P&O is used to catch the nearest LP and then, PSO is used to follow the dynamic GP. Nevertheless, obvious oscillations around steady state still exist in addition to the role of PSO which is ambiguous in the case of GP at the beginning where P&O can track the first peak regardless of whether it is LP or GP [17]. As a result, different methodologies of PSO re-initialization are proposed in order to track the dynamic GP. For example, some researches assumed that if the PV output power changes, the SP also changes [2, 4, 17–19]. Whereas, other researches [1, 6, 20] claimed that the SP will change based on the PV voltage and current variation either together [1, 20] or individually [6]. Finally, Sundareswaran *et al*. in [3] discovered that the SP changes based on the two constraints used in [1, 20] and [2, 4, 18, 19]. All previous methodologies in [1–4, 6, 17–20], do not guarantee that the SP changes due to either load variation, uniform radiations change, or even in uniform conditions. In addition, the SP may change while the search area may remain unchanged. Therefore, re-initialization of the particles is useless and may disturb the PV system without the need for that.

Based on the previous literature review, original PSO can track the GP under the same SP with undesirable oscillation around steady state. In addition, it cannot follow the dynamic GP under time variant SPs and sticks at the first GP. This paper proposes two new, efficient and accurate re-initialization methodologies of hybrid PSO-FLC to overcome the two previous shortcomings of PSO. Hybrid PSO-FLC not only catches the dynamic GP; but it also prevents the obvious oscillations around steady state when PSO is used individually. To the best of our knowledge, this research study is counted as the first study which discuss in detail, hybrid PSO-FLC and PSO with and without the re-initialization of the particles to track the dynamic GP continuously under time variant SPs. This paper is organized as follows; Section 2 describes the PV system modelling under PSC. Section 3 introduces the dynamic GP extraction based on hybrid PSO-FLC techniques with new and efficient methodologies of re-initialization. Section 4 presents and discusses the simulation results. Finally, Section 5 introduces conclusions and recommendations.

## 2. Modelling of the photovoltaic system under PSC

**Fig 1:**
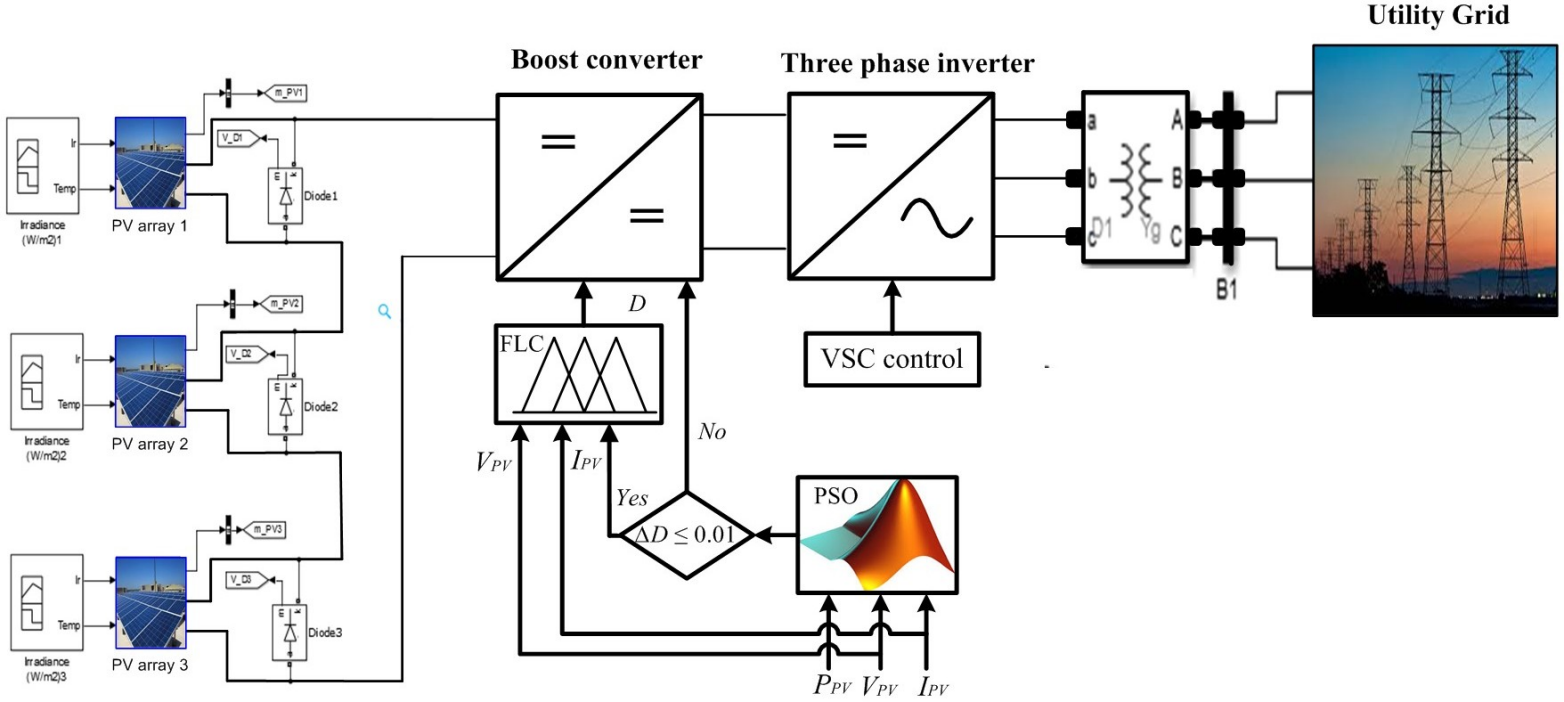
Schematic diagram of the PV system grid connected.

**Fig 2:**
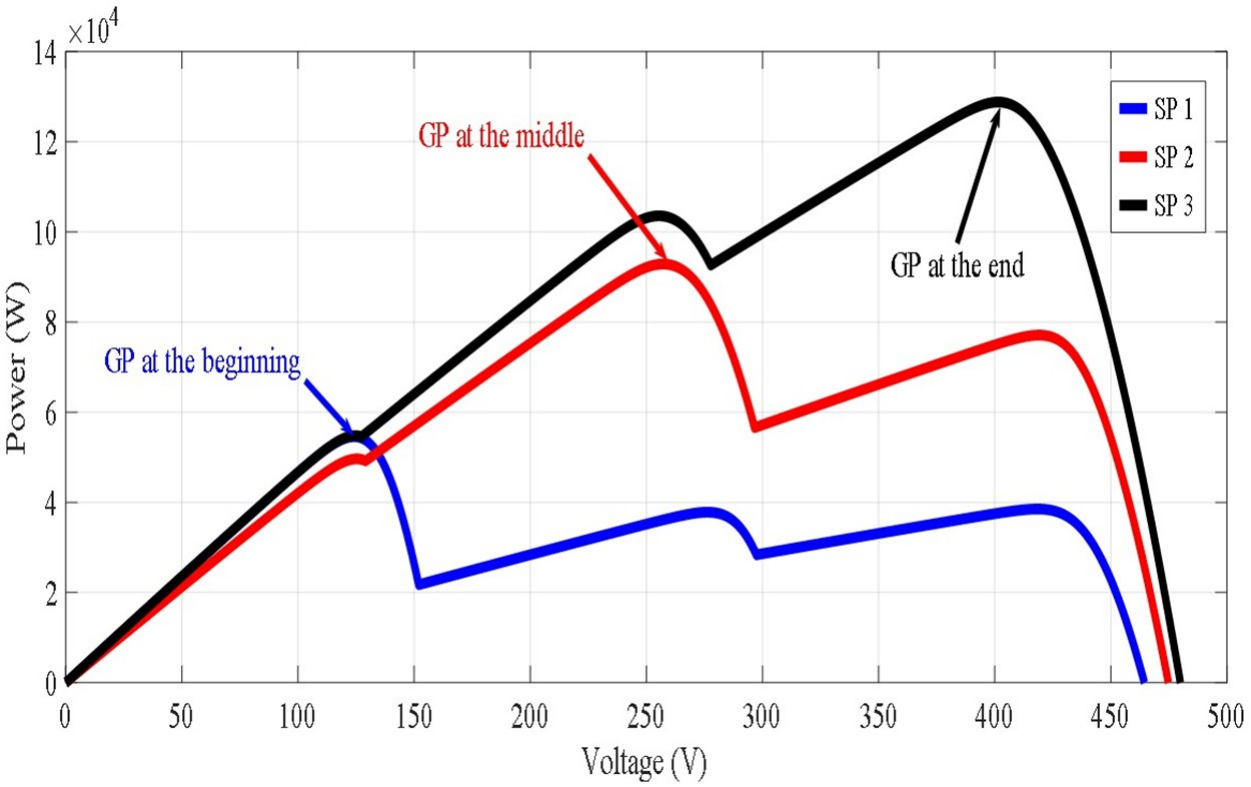
Three different shading patterns of the PV arrays under study.

Fig 1 shows the modelling of the PV system interconnected to the utility grid, which contains both the power and control circuits. The power circuit contains the PV array, boost converter and three-phase inverter interconnected to the utility grid. Whereas the control circuit includes PSO-FLC based MPPT and the three-phase inverter controller. Time variant radiations or SPs with three different values and positions of GP are applied. The time variant SPs are selected to guarantee the occurrence of the three available GP cases (GP at the beginning, GP at the middle, GP at the end) as shown in Fig 2. Time variant SPs are applied to generate a GP in different places on the P-V characteristics to see the output response of the hybrid PSO-FLC to follow the dynamic GP with and without dispersing the particles. Hybrid PSO-FLC aims to integrate between the merits of PSO and FLC. PSO can initially catch the dynamic GP under time variant SPs, but FLC cannot do this. On the other hand, FLC works conditionally to make soft tuning of the GP caught by PSO, which will be discussed in the next section. In addition, it prevents oscillations around steady state that occur when PSO is used alone. Co-simulation between M-file and Simulink is used to handle the simulation of the PV system shown in Fig 1.

## 3. Proposed hybrid PSO-FLC technique for dynamic global peak extraction

PSO is a time invariant MPPT technique. It can catch the GP under the same SP easily and accurately [7–13]. Once the SP changes, the GP’s position and value change and PSO cannot follow the new GP without dispersing the particles all over the search area. In addition, oscillations around steady state exist with PSO. Therefore, certain modifications on PSO is necessary to be a time variant GP tracking technique and deal with the time variant SP. In addition, another modification should be proposed to prevent oscillations around steady state. The logic steps of PSO without dispersing the particles are introduced in [21]. The particle’s velocity (*v*_*i*_^*k*^) and position (*x*_*i*_^*k*^) are updated using the following equations:

$$x_{i}^{k+1}=x_{i}^{k}+v_{i}^{k+1}$$ (1)

$$v_{i}^{k+1}=\omega v_{i}^{k}+c_{1}*P_{best\;i}+c_{2}*G_{best}-c_{3}x_{i}^{k}$$ (2)

In this paper, hybrid PSO-FLC is proposed to overcome the two previous shortcomings of PSO. As mentioned before, the integration between PSO and FLC results in the merits of PSO and FLC being combined together. Initially, PSO catches the initial value of the GP and its initial *G*_*best*_ (optimal duty ratio). Then, FLC makes soft tuning of the initial GP caught by PSO, which prevents oscillations around steady state that occur with PSO alone. Although, FLC cannot track the GP under PSC, it has many merits such as fast tracking speed, especially in the case of rapid changing atmospheric conditions, working properly even with an imprecise input data, fast and accurate convergence, high efficiency in tracking the first peak notwithstanding whether it is LP or GP compared to the conventional MPPT techniques [22–31]. FLC has two inputs;$\frac{dP_{PV}}{dV_{PV}}$ and $\Delta (\frac{dP_{PV}}{dV_{PV}})$ i.e. (*E*_*rr*_) and (Δ*E*_*rr*_) which can be determined from the PV output power and voltage as follows [32]:

$$E_{rr}=\frac{P_{PV}(k)-P_{PV}(k-1)}{V_{PV}(k)-V_{PV}(k-1)}$$ (3)

$$\Delta E_{rr}=E_{rr}(k)-E_{rr}(k-1)$$ (4)

**Fig 3:**
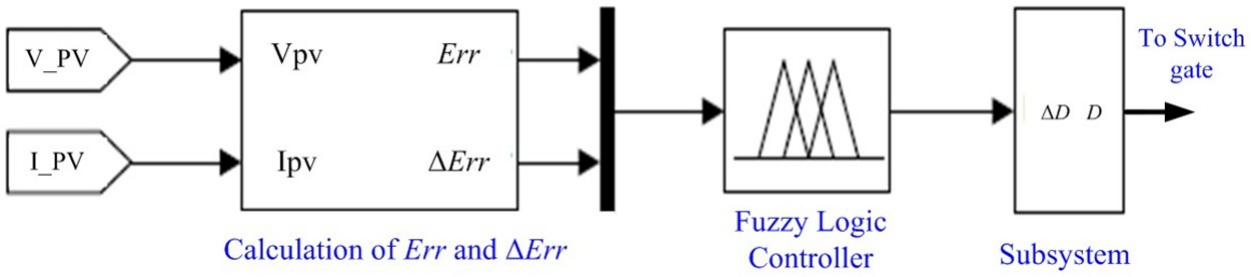
FLC block diagram in Simulink.

**Fig 4:**
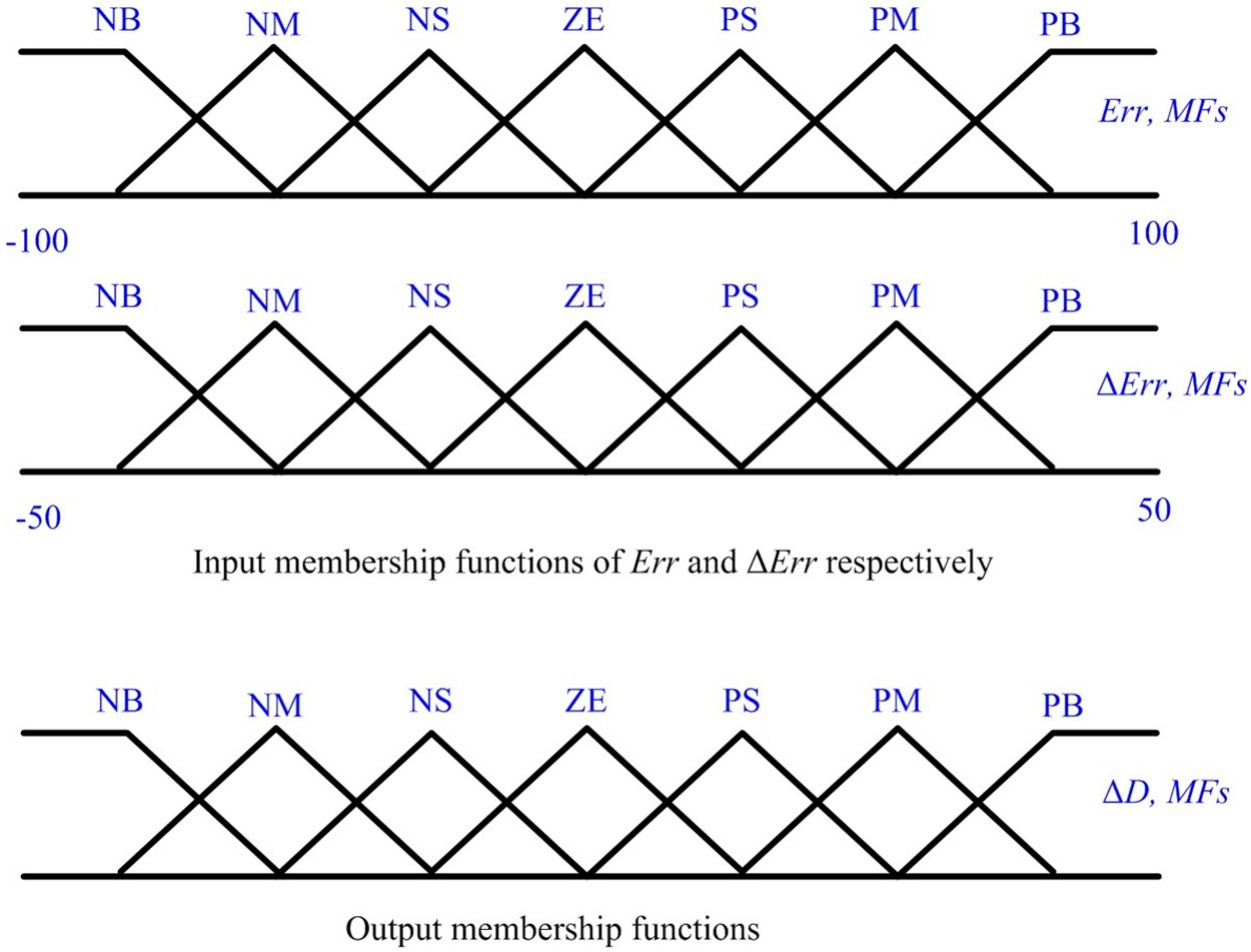
Membership functions of FLC.

The output from FLC is the required change in the duty ratio (Δ*D*), hence; the optimal duty ratio of the boost converter is determined. The FLC block diagram is shown in Fig 3. The inputs and output membership functions used in the simulation are shown in Fig 4. The variation step in Δ*E*_*rr*_ varies from -50 W to 50 W while it varies from -100W to 100W for *E*_*rr*_. The variation step of *E*_*rr*_ and Δ*E*_*rr*_ varies from the PV system to another. The membership names are introduced as follows: Positive Big (PB), Positive Medium (PM), Positive Small (PS), Zero (ZE), Negative Small (NS), Negative Medium (NM), Negative Big (NB) using basic fuzzy subset. Once *E*_*rr*_ and Δ*E*_*rr*_ are calculated and transferred to the logic variables based on the membership functions, the FLC output; *ΔD* of the boost converter; is estimated in rules. Therefore, the optimal duty ratio is determined.

The two new, efficient and accurate methodologies of PSO-FLC re-initialization are proposed to deal with the time variant SPs and catch the dynamic GP as follows:

### 3.1 PSO-FLC re-initialization upon predefined time

**Fig 5:**
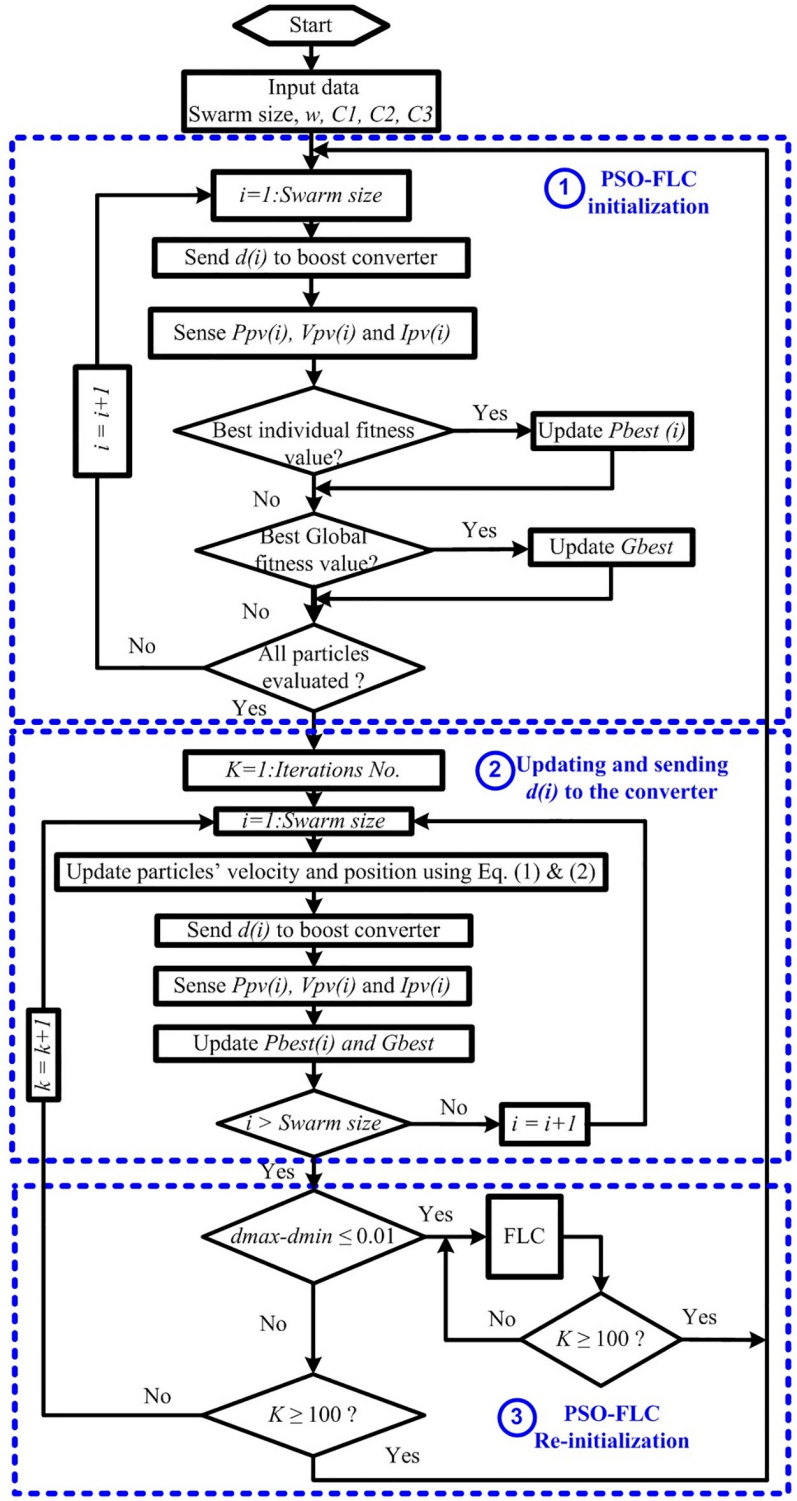
Flow chart of PSO-FLC re-initialization upon predefined time.

As discussed above, PSO can track the GP under the same SP. Once the SP changes, the position and value of the GP will also change. Therefore, PSO cannot catch the new GP and neither can it deal with the time variant SP because it is a time invariant MPPT technique. In addition, PSO-FLC without dispersing the particles cannot follow the dynamic GP under time variant SPs. To overcome this problem, the first new methodology of dispersing the PSO-FLC particles (re-initialization) is proposed upon certain predefined time (PDT) to make the particles research for the new GP in the whole search area. The PDT for re-initialization can be set depending on the SP frequency in the installation site. In this paper, the PDT is set to be 25 Sec (100 iterations). The flowchart of the PSO-FLC re-initialization upon predefined time is shown in Fig 5 and the logic steps of this methodology are shown in the following points:

- **Step 1** (PSO-FLC initialization parameters): Send the initial duty ratios (particles) [*d*_1_(0) *d*_2_(0) *d*_3_(0)] to the boost converter of the PV system (objective function) one by one and collect the corresponding powers.
- **Step 2** (Updating position and velocity of particles): update each particle’s position and velocity using the previously mentioned Eqs (1) and (2), respectively and get the new values of duty ratios [*d*_1_(i) *d*_2_(i) *d*_3_(i)].
- **Step 3** (Fitness evaluation): Send the new obtained duty ratios [*d*_1_(i) *d*_2_(i) *d*_3_(i)] to the boost converter and collect the corresponding power values [*P*_1_(i) *P*_2_(i) *P*_3_(i)]
- **Step 4** Evaluate the *P*_*best*,*i*_, *G*_*best*_ and their corresponding particle position (duty ratios). If the difference between *d*_*max*_ and *d*_*min*_ is less than or equal to 0.01, then go to FLC in the next step (Step 5); otherwise check if the time is greater than or if equal to PDT go to step 1; otherwise go to Step 2.
- **Step 5** (FLC): Check if the time is greater than or if equal to PDT, go again to Step 1; otherwise continue with FLC.

### 3.2 PSO-FLC Re-initialization upon shading pattern change

**Fig 6:**
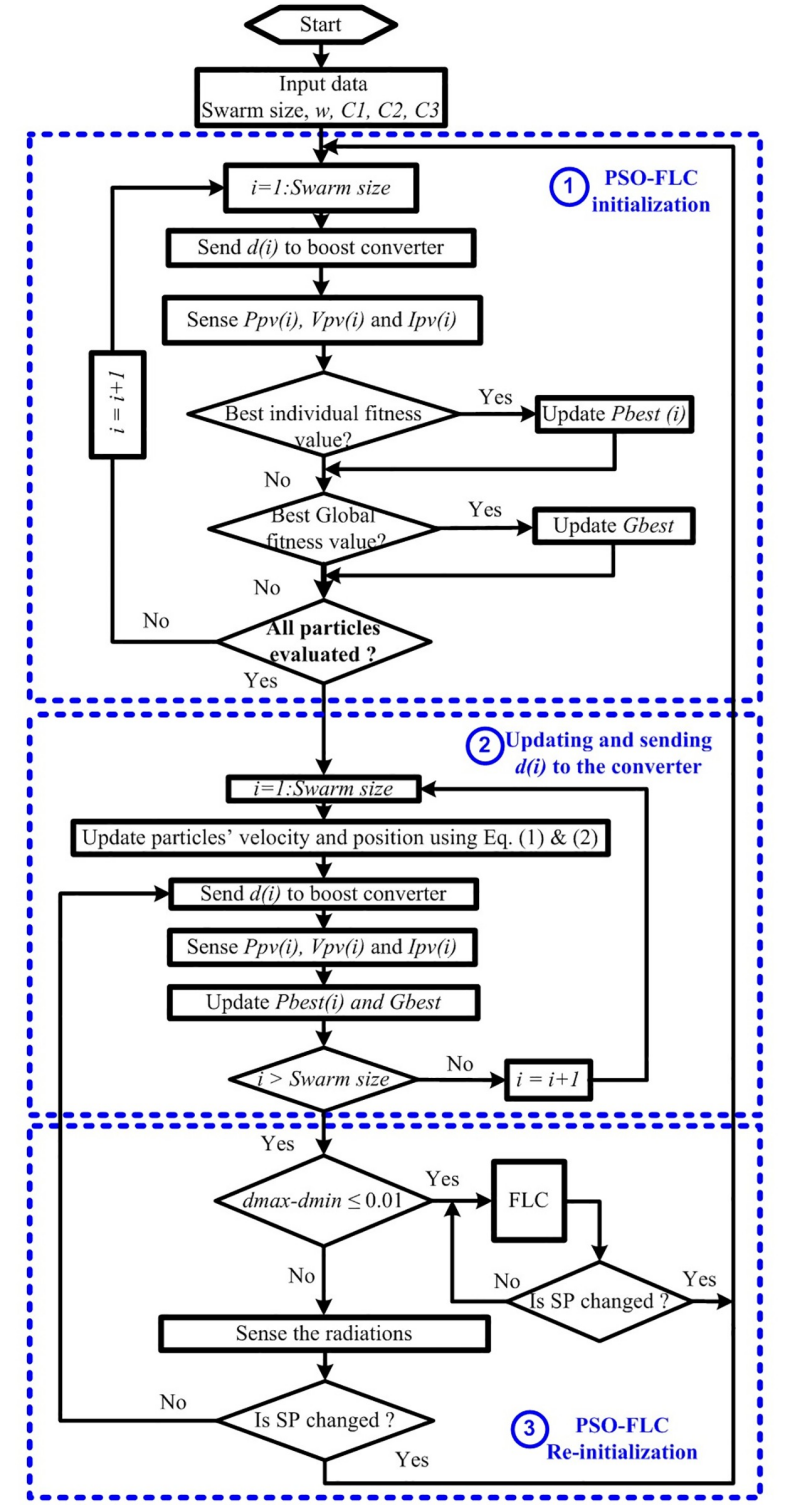
Flow chart of PSO-FLC re-initialization upon SP change.

The second methodology of dispersing the PSO-FLC particles (re-initialization) is proposed upon SP change. This will enable PSO-FLC to deal with the time variant SPs and follow the dynamic GP effectively and accurately. This methodology has many merits compared to the previous methodology such as high generated power captured and accurate re-initialization of the particles according to the need when SP changes. The flowchart of PSO-FLC re-initialization upon SP change is shown in Fig 6 and the logic steps of this methodology are shown in the following points:

- **Step 1** (PSO-FLC initialization parameters): Send the initial duty ratios (particles) [*d*_1_(0) *d*_2_(0) *d*_3_(0)] to the boost converter of the PV system (objective function) one by one and collect the corresponding powers.
- **Step 2** (Updating position and velocity of particles): Update each particle position and velocity using the previous mentioned Eqs (1) and (2), respectively and get the new values of duty ratio [*d*_1_(i) *d*_2_(i) *d*_3_(i)].
- **Step 3** (Fitness evaluation): Send the new obtained duty ratios [*d*_1_(i) *d*_2_(i) *d*_3_(i)] to the boost converter and collect the corresponding power values [*P*_1_(i) *P*_2_(i) *P*_3_(i)].
- **Step 4** Evaluate the *P*_*best*,*i*_, *G*_*best*_ and their corresponding particle position (duty ratios), then if the difference between *d*_*max*_ and *d*_*min*_ is less than or equal to 0.01, go to FLC in the next step (Step 5); otherwise, sense the radiation and temperature and if the SP has changed, go to Step 1; otherwise go to Step 2.
- **Step 5** (FLC): If the SP has changed, go to Step 1; otherwise continue with FLC.

Re-initialization of the PSO-FLC particles must be executed upon the change in SP in order to succeed in tracking the new GP in a new search area based on the proposed condition as follows:

$$\frac{G_{new}-G_{old}}{G_{old}}\geq \varepsilon$$ (5)

## 4. Simulation results and discussion

**Fig 7:**
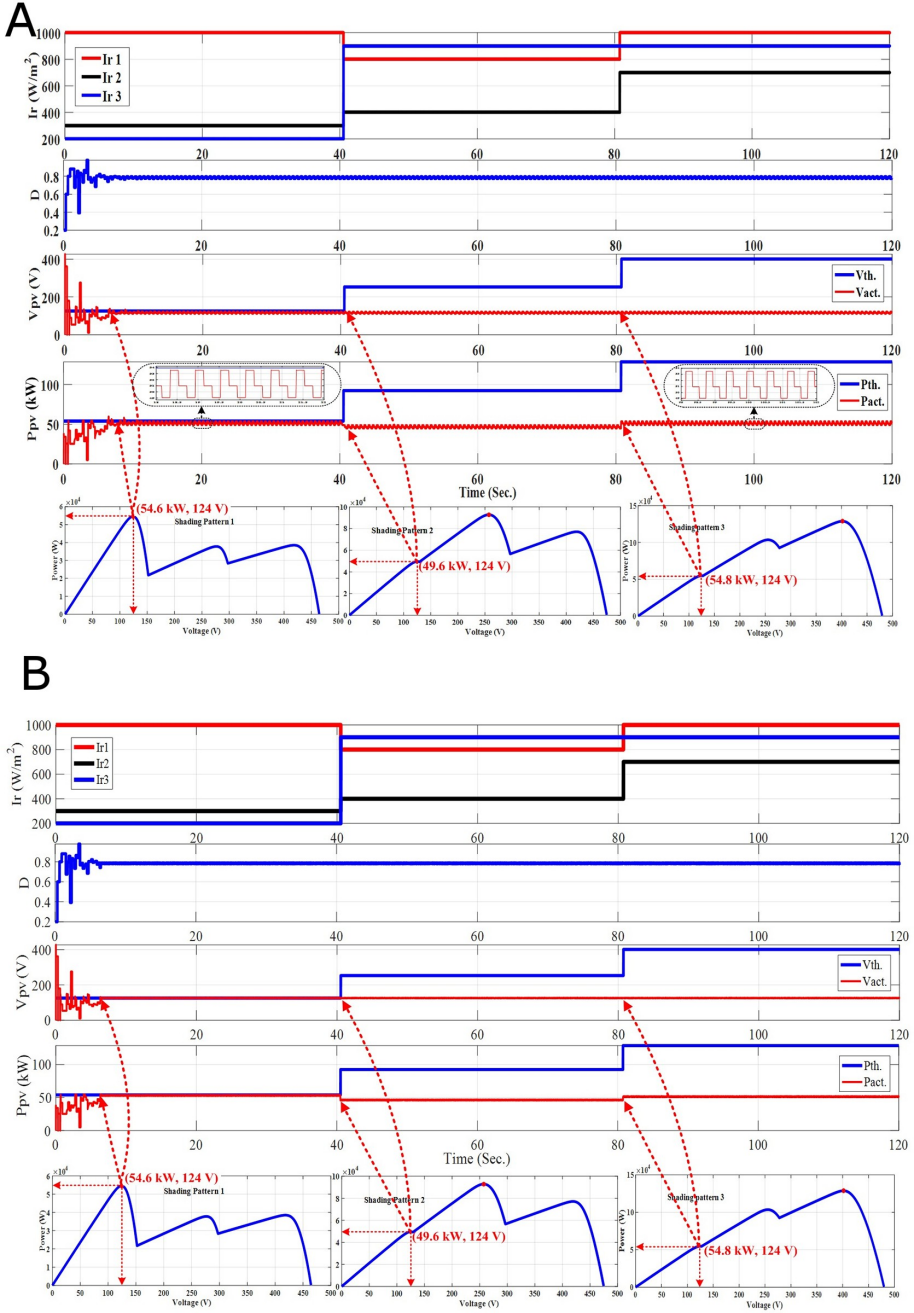
The PV output response for time variant SPs. (A) Standalone PSO without re-initialization. (B) Hybrid PSO-FLC without re-initialization.

The proposed hybrid PSO-FLC technique is compared to the original PSO to demonstrate the former’s more superior performance in tracking the dynamic GP and reducing the steady state oscillations. The power circuit of the PV system contains the PV array interconnected to the grid via boost converter and three-phase inverter. Whereas the control circuit contains the hybrid PSO-FLC algorithm. Three time variant SPs with three different GPs in terms of value and position are applied consequently. Each SP is applied for 40 Sec. with total simulation time of 120 Sec. for the three different SPs. The three corresponding GPs are GP at the beginning, GP at the middle and GP at the end as shown in Table 1 and Fig 7. The idea behind that is to study the effectiveness of the proposed hybrid PSO-FLC technique to track the dynamic GP under time variant SPs.

**Table 1:** Three shading patterns with three different cases of the GP.

SPs	Irradiance (W/m^2^)	SP1 (GP at the beginning)	SP2 (GP at the middle)	SP3 (GP at the end)
**Methodologies**	*G*_*1*_	1000	800	1000
	*G*_*2*_	300	400	700
	*G*_*3*_	200	900	900
	P_Th_* (kW)	54.6	92.8	128.8
	V_Th_* (V)	124	257	402
**PSO-FLC without re-initialization**	P_Act_*(kW)	54.6	49.6	54.8
	V_Act_* (V)	124	124	124
	Efficiency	100%	53.4%	42.6%
**PSO-FLC re-initialization at PDT**	P_Act_ (kW)	54.6	49.6 → 92.8	54.8 → 128.8
	V_Act_ (V)	124	124 → 257	257 → 402
	Efficiency	100%	53.4% → 100%	42.6% → 100%
**PSO-FLC re-initialization at SP change**	P_Act_ (kW)	54.6	92.8	128.8
	V_Act_ (V)	124	257	402
	Efficiency	100%	100%	100%

*P_Th_: Theoretical power; V_Th_: Theoretical voltage; P_Act_: Actual power; V_Act_: Actual voltage

On the other hand, three different SPs are applied in this study to prove that both PSO and hybrid PSO-FLC MPPT techniques without re-initialization cannot follow the dynamic GP. The purpose of re-initialization of PSO or PSO- FLC is to disperse the particles to deal with the new SP and new position of the GP. As a result, a detailed comparison between PSO and hybrid PSO-FLC with two new, efficient and accurate proposed methodologies of the particles re-initialization in addition to the original methodology without the particles re-initialization are discussed and analyzed as follow:

### 4.1 PSO-FLC without re-initialization (Case-1)

In this methodology, initialization the particles is executed once at the beginning. The PV system response for both PSO and PSO-FLC without re-initialization is introduced as follows:

- **Time interval from 0**–**40 Sec.:** Both PSO and PSO-FLC succeeded in tracking the first GP (54.6 kW) for SP1 as shown in Fig 7A and 7B. However, hybrid PSO- FLC has almost zero oscillations around steady state and makes soft tuning of the GP tracked compared to PSO alone.
- **Time interval from 40**–**80 Sec.:** The GP’s position and value changed (92.8 kW; GP at the middle) due to the variation of the SP (SP2). Nevertheless, both PSO and PSO-FLC cannot track the new GP and stick to the first GP position with a certain power-generated value, which is not LP or GP (49.6 kW). This means that both PSO and PSO-FLC cannot follow the dynamic GP upon SP change without the re-initialization of the particles to deal with the new SP and its new GP.
- **Time interval from 80**–**120 Sec.:** In a similarly, the GP’s position and value changed (128.8 kW; GP at the end) as a consequence of the SP change (SP3). Nevertheless, both PSO and PSO-FLC cannot track the new GP and stick to the first GP position with a certain power-generated value, which is not LP or GP (54.8 kW). This emphasizes the previous conclusion that both PSO and PSO-FLC cannot follow the dynamic GP upon SP change without the re-initialization of the particles to deal with the new SP and its new GP.

Although, hybrid PSO-FLC has a significant reduction of oscillations around steady state and makes soft tuning of the GP tracked compared to PSO alone, nevertheless, both PSO and hybrid PSO-FLC must be forced to disperse the particles to search for the new GP of the new SP occurred. In addition, both PSO and hybrid PSO-FLC without the re-initialization of the particles cannot track the new GP and stick to the first GP position with a certain power-generated value. The generated power value is not LP or GP, and equal to a certain power value related to the first GP duty ratio. Therefore, re-initialization of the particles must be executed upon one of the two new proposed methodologies of the PSO or hybrid PSO-FLC re-initialization that will be introduced, discussed and analyzed in the next sections.

### 4.2 PSO-FLC re-initialization upon predefined time (Case-2)

**Fig 8:**
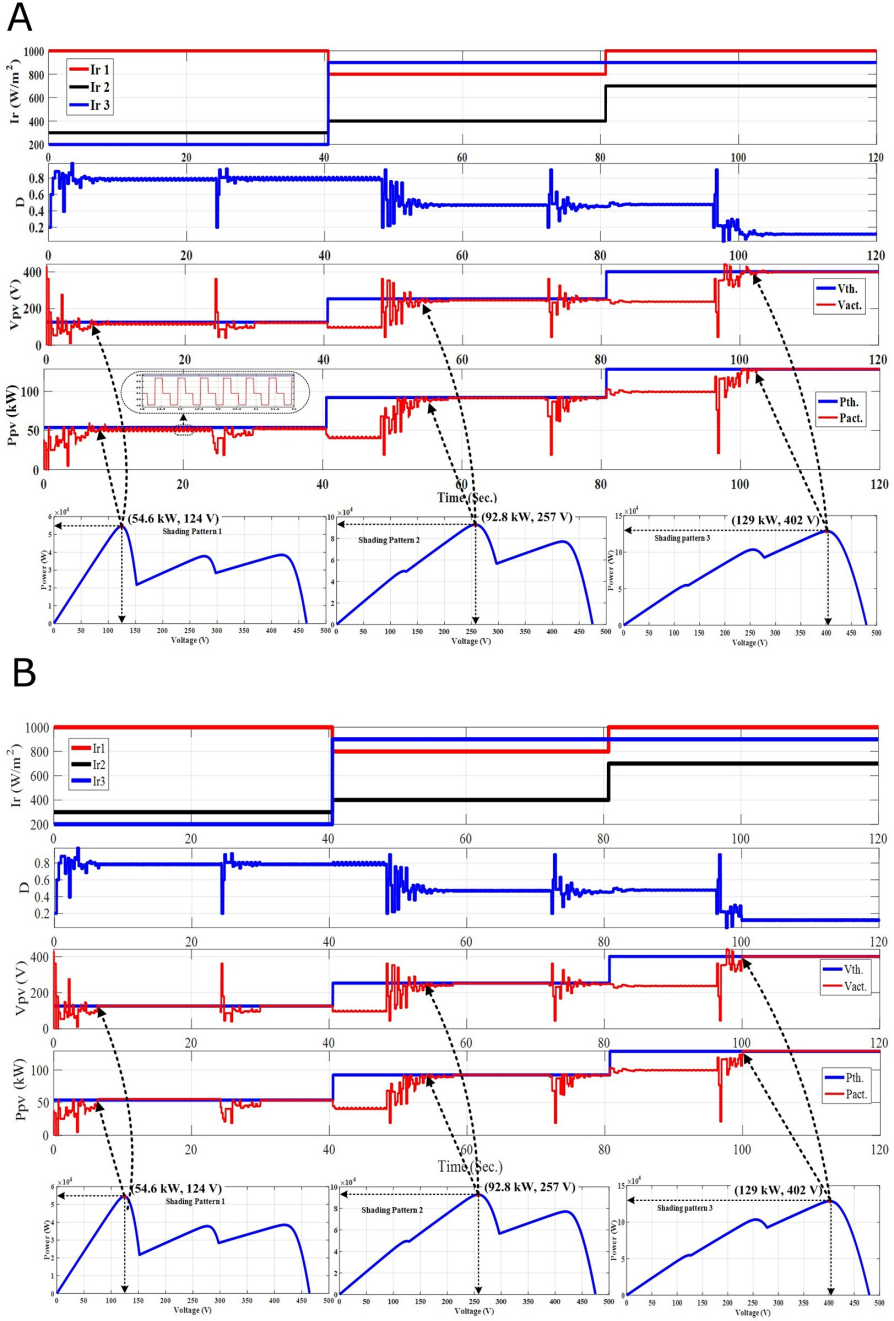
The PV output response for time variant SPs. (A) Standalone PSO re-initialization upon PDT. (B) Hybrid PSO-FLC re-initialization upon PDT.

As discussed above in case-1, PSO and hybrid PSO-FLC techniques without the re-initialization of the particles cannot track the variant GP and stick to the first GP position with a certain generated power value. PSO and hybrid PSO-FLC re-initialization upon predefined time (PDT) represents one of the new and efficient proposed methodologies of dispersing the particles to search for the new GP of the new SP in a new searching area. The PV system response of both PSO and PSO-FLC with re-initialization upon PDT (24 Sec.) is summarized as follows:

- **Time interval from 0**–**24 Sec.:** Both PSO and hybrid PSO-FLC succeeded in tracking the first GP (54.6 kW) for SP1 as shown in Fig 8A and 8B. However, hybrid PSO-FLC has zero almost oscillations around steady state and makes soft tuning of the GP tracked compared to PSO.
- **Time interval from 24**–**40 Sec.:** Re-initialization of PSO and hybrid PSO-FLC upon PDT (24 Sec.) is carried out for the same SP. Therefore, the PV system works at the same operating point (54.6 kW).
- **Time interval from 40**–**48 Sec.:** The GP’s position and value change (GP at the middle; 92.8 kW) as a consequence of the change in SP (SP2). Nevertheless, both PSO and hybrid PSO-FLC cannot track the new GP until the re-initialization of PSO and PSO-FLC is executed.
- **Time interval from 48**–**72 Sec.:** Re-initialization of PSO and hybrid PSO-FLC is executed at the beginning of this interval. Therefore, they succeeded in tracking the new GP (92.8 kW) at *d* = 0.43.
- **Time interval from 72**–**80 Sec.:** Re-initialization of PSO and hybrid PSO-FLC is carried out at the beginning of this interval for the same SP. Therefore, the PV system works at the same operating point (92.8 kW).
- **Time interval from 80**–**96 Sec.:** The GP’s position and value change (GP at the end; 129 kW) as a consequence of the change in SP (SP3). Nevertheless, both PSO and PSO-FLC cannot track the new GP until the re-initialization of PSO and PSO-FLC is executed.
- **Time interval from 96**–**120 Sec.:** Re-initialization of PSO and hybrid PSO-FLC is executed at the beginning of this interval. Therefore, they succeeded to track the new GP of SP3 (129 kW).

Under time variant SPs, both PSO and hybrid PSO-FLC re-initialization upon PDT have many benefits such as simple in implementation, no need for temperature and radiation sensors, avoidance of sensor problems and improvement of the PV system performance. Hybrid PSO-FLC has outperformed PSO in making fine-tuning of the GP tracked and reducing oscillations around steady state effectively. The unique problem of this new methodology is the delayed response with the SP change (not simultaneously).

### 4.3 PSO-FLC re-initialization upon the SP change (Case-3)

**Fig 9:**
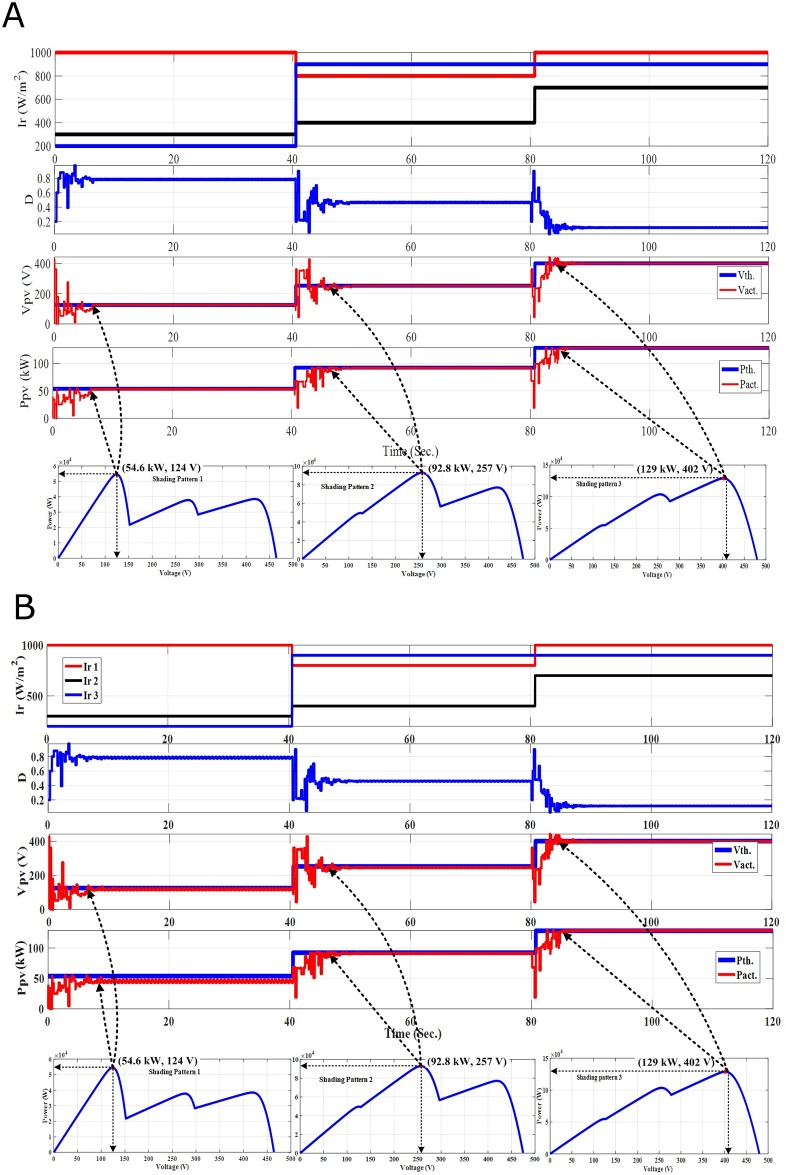
The PV output response for time variant SPs. (A) Standalone PSO re-initialization upon SP change. (B) Hybrid PSO-FLC re-initialization upon SP change.

In Case-1, PSO and hybrid PSO-FLC techniques without re-initialization cannot track the dynamic GP under time variant SPs. Whereas a simple methodology is proposed in Case-2 to disperse the particles (re-initialization) upon PDT and it succeeded in following the dynamic GP; but, with a delayed response or unwanted re-initialization. Finally, an accurate and efficient methodology (Case-3) is proposed to re-initialize the particles upon SP change to deal with the time variant SPs and follow the dynamic GP. The PV system response of both PSO and hybrid PSO-FLC with re-initialization upon SP change is summarized as follows:

- **Time interval from 0**–**40 Sec.:** Both PSO and PSO-FLC succeeded to catch the first GP of SP1 (54.6 kW) as shown in Fig 9A and 9B. Hybrid PSO-FLC reduces the occurrence of oscillations around steady state that is the case with PSO and fine-tune of the GP tracked.
- **Time interval from 40**–**80 Sec.:** The GP’s position and value changed (GP at the middle; 92.8 kW) due to the variation of the SP (SP2). Both PSO and PSO-FLC re-initialization upon the SP change at t = 40 Sec. were carried out and they succeeded in catching the second GP (92.8 kW) as shown in Fig 9A and 9B.
- **Time interval from 40**–**80 Sec.:** In similar, the GP’s position and value is changed GP at the end; 129 kW) due to the variation of the SP (SP3). Both PSO and PSO-FLC re-initialization upon the SP change at t = 80 Sec has been carried out and they succeeded to catch the second GP (129 kW) as shown in Fig 9A and 9B, but PSO-FLC works at GP with almost zero oscillations as shown in Fig 9B.

Based on the above analysis and discussions, the methodology of dispersing the particles (re-initialization) upon the SP change represents the most accurate and efficient methodology to deal with the time variant SP. It overcame the shortcomings of the previous methodologies where it can track the dynamic GP effectively compared to other proposed methodologies. In addition, it performed well and the system efficiency improved considerably in all PSCs as shown in Table 1. Although the radiation sensors will help us to detect the SP change that facilitate the re-initialization decision, it will add additional cost to the total costs and system’s disturbance occur during cloudy days.

## 5. Conclusions

PSO is a time invariant MPPT technique. It can track the GP under the same SP effectively. Nevertheless, obvious oscillations around steady state exist. In addition, it cannot follow the dynamic GP under time variant SPs and stick at the first GP position with a certain power-generated value that is not GP. Therefore, two new proposed methodologies of dispersing the particles (re-initialization) are implemented with PSO and hybrid PSO-FLC to deal with the time variant SPs and follow the dynamic GP. Although, PSO and hybrid PSO-FLC with re-initialization show a more superior performance in dispersing the particles and ability to follow the dynamic GP under time variant SPs, nevertheless, the re-initialization methodology upon the SP change has outperformed the other re-initialization one upon PDT in terms of tracking speed, time response, efficiency and accuracy. On the other hand, Hybrid PSO-FLC collects both the merits of PSO and FLC. The integration purpose between PSO and FLC technique is not only to follow the dynamic GP but also to reduce the obvious oscillations around steady state that occur with PSO alone. Hybrid PSO-FLC based GP tracking performs well compared to PSO in terms of efficiency, accuracy, oscillations reduction around steady state and fine-tuning of the GP tracked.
